# Effects of Whey, Soy or Leucine Supplementation with 12 Weeks of Resistance Training on Strength, Body Composition, and Skeletal Muscle and Adipose Tissue Histological Attributes in College-Aged Males

**DOI:** 10.3390/nu9090972

**Published:** 2017-09-04

**Authors:** C. Brooks Mobley, Cody T. Haun, Paul A. Roberson, Petey W. Mumford, Matthew A. Romero, Wesley C. Kephart, Richard G. Anderson, Christopher G. Vann, Shelby C. Osburn, Coree D. Pledge, Jeffrey S. Martin, Kaelin C. Young, Michael D. Goodlett, David D. Pascoe, Christopher M. Lockwood, Michael D. Roberts

**Affiliations:** 1 School of Kinesiology, Auburn University, Auburn, AL 36849, USA; moblecb@auburn.edu (C.B.M.); cth0023@auburn.edu (C.T.H.); par0021@auburn.edu (P.A.R.); pwm0009@auburn.edu (P.W.M.); mzr0049@auburn.edu (M.A.R.); wck0007@auburn.edu (W.C.K.); rga0001@auburn.edu (R.G.A.); cgv0001@auburn.edu (C.G.V.); sco0004@auburn.edu (S.C.O.); cdp0017@auburn.edu (C.D.P.); jmartin@auburn.vcom.edu (J.S.M.); kyoung@auburn.vcom.edu (K.C.Y.); pascodd@auburn.edu (D.D.P.); 2 Department of Cell Biology and Physiology, Edward via College of Osteopathic Medicine—Auburn Campus, Auburn, AL 36832, USA; goodlmd@auburn.edu; 3 Athletics Department, Auburn University, Auburn, AL 36849, USA; 4 Lockwood, LLC, Draper, UT 84020, USA; chris@drchrislockwood.com

**Keywords:** satellite cell, resistance training, leucine, whey, soy

## Abstract

We sought to determine the effects of L-leucine (LEU) or different protein supplements standardized to LEU (~3.0 g/serving) on changes in body composition, strength, and histological attributes in skeletal muscle and adipose tissue. Seventy-five untrained, college-aged males (mean ± standard error of the mean (SE); age = 21 ± 1 years, body mass = 79.2 ± 0.3 kg) were randomly assigned to an isocaloric, lipid-, and organoleptically-matched maltodextrin placebo (PLA, *n* = 15), LEU (*n* = 14), whey protein concentrate (WPC, *n* = 17), whey protein hydrolysate (WPH, *n* = 14), or soy protein concentrate (SPC, *n* = 15) group. Participants performed whole-body resistance training three days per week for 12 weeks while consuming supplements twice daily. Skeletal muscle and subcutaneous (SQ) fat biopsies were obtained at baseline (T1) and ~72 h following the last day of training (T39). Tissue samples were analyzed for changes in type I and II fiber cross sectional area (CSA), non-fiber specific satellite cell count, and SQ adipocyte CSA. On average, all supplement groups including PLA exhibited similar training volumes and experienced statistically similar increases in total body skeletal muscle mass determined by dual X-ray absorptiometry (+2.2 kg; time *p* = 0.024) and type I and II fiber CSA increases (+394 μm^2^ and +927 μm^2^; time *p* < 0.001 and 0.024, respectively). Notably, all groups reported increasing Calorie intakes ~600–800 kcal/day from T1 to T39 (time *p* < 0.001), and all groups consumed at least 1.1 g/kg/day of protein at T1 and 1.3 g/kg/day at T39. There was a training, but no supplementation, effect regarding the reduction in SQ adipocyte CSA (−210 μm^2^; time *p* = 0.001). Interestingly, satellite cell counts within the WPC (*p* < 0.05) and WPH (*p* < 0.05) groups were greater at T39 relative to T1. In summary, LEU or protein supplementation (standardized to LEU content) does not provide added benefit in increasing whole-body skeletal muscle mass or strength above PLA following 3 months of training in previously untrained college-aged males that increase Calorie intakes with resistance training and consume above the recommended daily intake of protein throughout training. However, whey protein supplementation increases skeletal muscle satellite cell number in this population, and this phenomena may promote more favorable training adaptations over more prolonged periods.

## 1. Introduction

There is widespread evidence suggesting that protein supplementation enhances resistance training adaptations. For instance, Cribb et al. [1] reported that resistance trained participants who consumed 45 g/day of whey protein isolate following 10 weeks of resistance training achieved a 5 kg increase in lean body mass (LBM) which was 4.2 kg greater than a casein-supplemented group. Burke et al. [2,3] reported that whey protein supplementation promoted 2.3–2.5 kg increases in LBM with 6 weeks of resistance training which was ~1.4 kg greater than the effects observed in these studies’ maltodextrin placebo (PLA) groups. Notably, a soy protein group was also examined in one of the two aforementioned studies [3], and this group experienced a 1.7 kg increase in LBM which was not statistically greater than the PLA group. Hulmi et al. [4] reported that 10.5 weeks of resistance training and whey protein supplementation elicited a 2.5 kg increase in LBM which was 0.5 kg greater than the non-energetic PLA group. Furthermore, Volek et al. [5] reported that 9 months of resistance training combined with whey or soy protein supplementation resulted in 3.6 and 2.6 kg increases in LBM, respectively. While the aforementioned studies did not determine the mechanisms responsible for the reported phenotypic changes in skeletal muscle mass, others have postulated that protein supplementation reduces fast to slow isoform shifts [6] and promotes myogenic responses to resistance training [7]. A recent review by Morton et al. [8] provides additional studies reporting that protein supplementation in conjunction with resistance training enhances indices of skeletal muscle anabolism, although other studies have reported that protein supplementation has no added benefit when performed in conjunction with 8–12 weeks of resistance training [9,10]. 

Contrary to much of the positive data supporting whey protein supplementation, the data appears to be less favorable regarding the effects of amino acid-only supplementation in enhancing resistance training induced increases in LBM. For instance, Bird et al. [11] reported that essential amino acid (EAA) supplementation (6 g/day) led to a ~3 kg increase in LBM following 12 weeks of resistance training twice a week in younger untrained males, albeit these increases were not significantly different from a PLA group. Vieillevoye et al. [12] reported similar findings in younger untrained males whereby 15 g/day of EAA supplementation during 12 weeks of resistance training did not increase LBM compared to a sucrose PLA group. Additionally, Aguilar et al. [13] recently reported that younger male subjects who supplemented with L-leucine (LEU; 3 g/day) during 8 weeks of resistance training experienced no additional increase in quadriceps muscle size increases when compared to subjects consuming a cornstarch PLA. 

In spite of the aforementioned literature suggesting that whey or soy protein supplementation may be more effective than EAA or LEU in promoting additional increases in LBM with resistance training, a prevailing hypothesis is that the LEU content of a given dietary protein determines the efficacy of how that protein potentiates muscle growth. However, this hypothesis is based on acute human, animal, or cell culture-based studies reporting that LEU or whey protein (which contains 8–11% LEU) optimally stimulates muscle protein synthesis [14,15,16,17]. To this end, there is no evidence which has directly compared the anabolic effects of LEU supplementation versus supplementation with other dietary protein sources that contain high levels of LEU (e.g., whey or soy). Therefore, the purpose of this study was to examine if supplementation with LEU, whey protein concentrate (WPC), whey protein hydrolysate (WPH) or soy protein concentrate (SPC) enhances markers of skeletal muscle hypertrophy with resistance training in previously untrained, college-aged males. A secondary aim was to also assess how these different supplements affected subcutaneous (SQ) fat cell size from biopsy specimens given that recent data from our group has demonstrated that WPH can elicit lipolytic effects [10,18,19]. Notably, the servings from all supplement groups (except the maltodextrin placebo described below) were standardized for LEU content (~3 grams per serving) and ingested twice daily. Based upon the supporting literature, we hypothesized that individuals consuming whey protein supplements would experience greater increases in indices related to muscle anabolism compared to those consuming the LEU, SPC and PLA supplements.

## 2. Materials and Methods

### 2.1. Ethical Approval and Screening of Participants

Prior to initiating this study, the protocol was reviewed and approved by the Auburn University Institutional Review Board (IRB), and was conducted in accordance with the Declaration of Helsinki (approved protocol #: 15-320 MR 1508; IRB contact: irbadmin@auburn.edu). Healthy, untrained, college-aged male (i.e., 19–23) participants were recruited for this study. All enrolled participants provided verbal and written study consent, completed a medical history form, and were given a 4-day food log to complete prior to initiating the study. These screening forms ensured that all eligible participants were healthy and recreationally active but (a) had not engaged in any regular exercise program for at least 6 months prior to study initiation (≤2 resistance training exercise or high-intensity aerobic exercise sessions/week); (b) were not currently consuming a high-protein diet (≥2.0 g/kg/day); (c) were not using anabolic enhancing agents (e.g., anabolic steroids, supplemental protein, creatine monohydrate, or prohormones); or (d) did not have medical or orthopedic condition(s) that would hinder them from participating in the current study. Once initial screening was complete, all eligible participants were scheduled to return to the School of Kinesiology at Auburn University one week later for baseline testing (T1). 

### 2.2. Study Design

**Figure 1:**
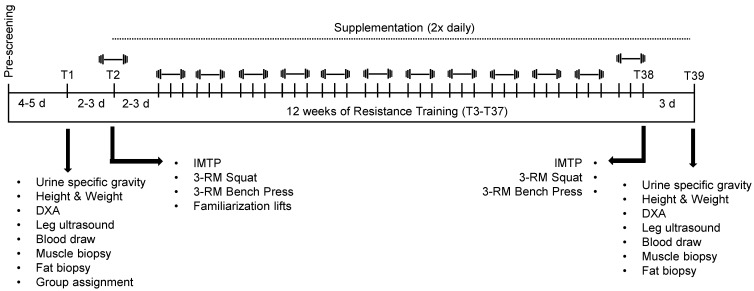
Study design. Abbreviations: DXA, dual x-ray absorptiometry; 3-RM, three-repetition maximum test; IMTP, isometric mid-thigh pull.

The study design implemented was double-blinded and placebo-controlled (Figure 1). Likewise, we followed guidelines established by the CONSORT Transparent Reporting of Trials established in 2010, and this trial was registered at ClinicalTrials.gov (unique protocol ID: 15-320 MR 1508). Participants were encouraged to refrain from rigorous physical activity for 4–5 days prior to baseline testing (T1). For T1, participants were instructed to report to the laboratory in a well-hydrated, 4-h fasted state whereby they were subjected to the following assessments: (a) urine specific gravity; (b) height and body mass; (c) body composition using dual-energy X-ray absorptiometry (DXA) (General Electric Lunar Prodigy enCORE, software version 10.50.086; Madison, WI, USA); (d) vastus lateralis thickness using ultrasonography (General Electric LOGIQ S7 Expert; Chicago, IL, USA); (e) venipuncture; (f) percutaneous skeletal muscle biopsy collection from the vastus lateralis; and (g) a percutaneous SQ fat biopsy from the gluteal region. Two to three days following T1, subjects reported back to the laboratory in a 4-h fasted state for a second visit (T2) whereby maximal force production capacity was assessed using an isometric mid-thigh pull (IMTP) test, lower body strength was assessed using a three repetition maximum (3-RM) squat, and upper body strength was assessed using a 3-RM bench press. Additionally, during T2, subjects were familiarized with all lifts that were to be performed during the training intervention. Following T2, subjects engaged in 12 weeks of resistance training and supplementation. The last training bout (T38) consisted of IMTP as well as squat and bench press 3-RM re-assessments in a 4-h fasted state. Seventy-two hours following T38, subjects reported back to the laboratory in a 4-h fasted state for post-testing (T39) which consisted of all body composition, and blood and biopsy collection procedures noted for T1. All of the aforementioned testing procedures as well as training and supplementation procedures are described in greater detail below.

### 2.3. Body Composition Testing

During T1 and T39, participants were instructed to submit a urine sample (~5 mL) to assess normal hydration specific gravity levels (1.005–1.020 ppm) using a handheld refractometer (ATAGO; Bellevue, WA, USA). Participants with a urine specific gravity ≥1.020 were asked to consume tap water every 15 min for 30 min and then were re-tested. Following hydration testing, height and body mass were assessed using a digital column scale (Seca 769; Hanover, MD, USA) with weights and heights collected to the nearest 0.1 kg and 0.5 cm, respectively. Next, participants were subjected to a full body DXA scan while wearing general sports attire (i.e., athletic shorts or compression shorts and an athletic shirt) to assess various body composition characteristics. Dual arm and dual leg lean muscle mass, as assessed by the accompanying software, were used to estimate total body skeletal muscle mass (TBMM) by employing the equation from Kim et al. [20], as reported by our group previously [10]. Notably, body segmentation for each scan was standardized prior to analyses by the same technician. Total body fat mass was also assessed by the accompanying software. According to previous data published by our laboratory, the same-day reliability of the DXA during a test-calibrate-retest on 10 participants produced intra-class correlation coefficients of 0.998 for total body fat mass (mean difference between tests = 0.40 ± 0.05 kg), 0.998 for total body lean mass (mean difference between tests = 0.29 ± 0.13 kg), and 0.998 for dual-leg lean mass (mean difference between tests = 0.17 ± 0.09 kg) [21].

Following DXA scans, participants were subjected to an ultrasound assessment to determine vastus lateralis muscle thickness. Measurements were taken from the midway point between the iliac crest and patella of the right femur whereby subjects were in a standing position and all weight was placed on the left leg. All DXA scans and ultrasound assessments were completed by the same investigator as suggested by previous research interventions [10,22] in order to minimize variability in testing procedures.

### 2.4. Venipuncture, and Percutaneous Skeletal Muscle and Fat Biopsies

T1 and T39 venous blood samples were aseptically collected from the antecubital vein and collected into a 5 mL serum separator tube (BD Vacutainer; Franklin Lakes, NJ, USA). Notably, this blood was saved for further experimentation and variables assessed from these blood draws are not presented herein. Immediately following blood collection, participants were instructed to lay in a supine position on a treatment table whereby a percutaneous skeletal muscle biopsy was aseptically obtained from the left vastus lateralis muscle using a 5 gauge Bergstöm needle with suction as previously described by our laboratory [23,24,25,26]. Approximately, 20–40 mg of skeletal muscle tissue for each time point was placed in a cryomold with OCT media (Electron Microscopy Sciences; Hatfield, PA, USA). Cryomolds were then slow-frozen in liquid-nitrogen-cooled isopentane and stored at −80 °C for immunohistochemistry analyses that are described below. Sections of SQ fat (1–2 cm) extracted from the gluteal aspect of the left hip were placed in 10% formalin and preserved for hematoxylin and eosin (H&E) staining and histological analyses which are described in detail below. Following T1 testing procedures, subjects were counterbalanced into one of five groups based upon DXA LBM values in order to ensure that baseline values did not differ between supplement groups. More details regarding supplementation are described below, and supplementation began immediately following the first training bout (T3).

### 2.5. Isometric Mid-Thigh Pull, Strength Testing, and Weightlifting Familiarization

During T2 and T38, participants were instructed to report back to the laboratory under well hydrated, 4-h fasted conditions for strength testing and weight training familiarization (T2). First, each participant completed an IMTP test which has been validated to approximate whole-body maximal voluntary strength [27,28,29]. Briefly, knee and hip angles (125° ± 5° and 175° ± 5°, respectively) were measured using a standard goniometer (Fabrication Enterprises; White Plains, NY, USA). A standard, 20 kg barbell (York Barbell; York, PA, USA) and STS Power Rack (York Barbell) were used to conduct the IMTP. Dual OR6-7 force plates (AMTI; Watertown, MA, USA) with dual Gen 5 amplifiers (AMTI) sampling at 500 Hz were used to measure vertical force production in Newtons (N). Similar to other investigations [29,30,31,32], each participant was allowed at least two attempts, and up to four attempts if differences in vertical peak force between trials were >250 N. Manufacturer software was used to calculate vertical peak force during the testing sessions, and a custom-written MATLAB script (Natick, MA, USA) was employed to identify the greatest vertical force produced in N for each trial, post-hoc. Two trials within 250 N were used to calculate an average vertical peak force across trials and functioned as a metric for maximal voluntary force production (i.e., strength) in this investigation. 

Approximately 5 min following T2 and T38 IMTP testing, participants performed 3-RM back squat and bench press assessments using a 20 kg barbell (York Barbell), STS Power Rack (York Barbell) and free weights. The demonstration of proper technique as well as the implementation of progressively-loaded 3-RM tests were overseen by C.B.M. and C.T.H. who possess the Certified Strength and Conditioning Specialist (CSCS) credential from the National Strength and Conditioning Association (NSCA). Bench press and back squat repetitions were considered to be successful when performed through the full range of motion (i.e., chest touch to full arm extension for bench press, and eccentric lowering past 90° knee flexion for back squat). A repetition was not counted if subjects exhibited poor and/or unsafe technique or needed assistance with a repetition during maximal testing.

Approximately 5 min following T2 3-RM testing, participants were instructed to perform the other two major lifts that were implemented for training (i.e., deadlift and bent-over-row) in the presence of CSCS-certified personnel. The goal of this session was to familiarize each participant with appropriate lifting technique in order to minimize the risk of injury throughout the course of the study. 

### 2.6. Training Protocol

For visits 3–37 (T3–T37), a daily undulating periodization (DUP) training model was employed over the 12-week training period given that this model has been shown to be more beneficial in eliciting greater increases in strength [33,34] and hypertrophy [35,36] than traditional linear periodization training models. Specifically, participants were instructed to perform free-weighted barbell squats, bench press, deadlifts, and bent-over-rows for 4 sets of 10 repetitions (Monday or Tuesday), 6 sets of 4 repetitions (Wednesday or Thursday), and 5 sets of 6 repetitions (Friday or Sunday). Immediately following each completed set, a rating of perceived exertion (RPE) score was acquired from each participant (scale: 1–10) in order to monitor and progress of each participant accordingly while minimizing the potential risk of injury [37,38,39,40]. The RPE scale was described to participants as the remaining number of repetitions that the participant would be able to complete while employing good technique (i.e., 1 = 9 remaining repetitions in reserve, 10 = 0 remaining repetitions in reserve). More information on relative training intensities and progression can be found in Table 1. Participants were instructed to attend all 36 resistance training sessions throughout the duration of the study, but those that missed more than 4 sessions were not included in the analysis due to lack of training compliance. All participants were supervised by laboratory personnel for each training session to ensure that proper lifting technique was executed, and training volumes for each session were recorded. 

### 2.7. Supplementation

As stated above, participants were assigned to ingest either a PLA, LEU, WPC, WPH, or SPC supplement throughout the training intervention. More information regarding the macronutrient profile for a serving size of each supplement can be found in Table 2. On training days (T3–T37), participants consumed an individually-packaged serving in ~500 mL of tap water immediately following each training session under direct observation of the study personnel. Additionally, participants were instructed to consume an individual serving within 30 min prior to bedtime on training days given that this strategy has been shown to be effective for stimulating overnight muscle protein synthesis [41]. On non-training days, participants were instructed to consume an individual serving between a meal of their choosing and 30 min prior to bedtime. Supplements were separated into individual ready-made supplement-coded packets for daily consumption, and participants were given a 3-week supply. Study personnel collected and counted empty packets from each participant every 3 weeks before the next 3-week supply was distributed. Participants that did not consume ≥80% were not included in the analysis due to lack of compliance. 

Each supplement, except PLA, was formulated to provide ~3 g of leucine, per serving. Furthermore, each supplement was formulated to yield similar amounts of total energy (kcal) and fat (g), and was double-blinded to laboratory personnel and participants for group, appearance, taste, texture, and packaging. The WPC supplement was formulated using an agglomerated, 80% WPC (Hilmar™ 8010, Hilmar Ingredients; Hilmar, CA, USA). The WPH supplement was formulated using an agglomerated, partially hydrolyzed (12.5% degree of hydrolysate (12.5% DH), yielding approximately 67% of peptides as <5 kilodaltons (kDa) in molecular weight) 80% whey protein concentrate (Hilmar™ 8360, Hilmar Ingredients); SPC used an agglomerated, 80% soy protein concentrate (ALPHA^®^ 5812, Solae, LLC; St. Louis, MO, USA); LEU used an agglomerated, L-Leucine (L-Leucine USP, Glambia Nutritionals; Carlsbad, CA, USA) and non-GMO, corn-derived maltodextrin (MALTRIN^®^-M100; Grain Processing Corporation; Muscantine, IA, USA); and, the PLA group was formulated using maltodextrin (MALTRIN^®^-M100; Grain Processing Corporation). All five supplements were manufactured at JW Nutritional, LLC (Allen, TX, USA), a United States Food and Drug Administration cGMP-compliant facility independently audited and pre-qualified by Obvium*Q, LLC (Phoenix, AZ, USA), a GMP regulatory compliance firm. Personnel at JW Nutritional, LLC and C.M.L. (Lockwood, LLC; Draper, UT, USA) formulated and maintained blinding of groups, and each supplement was assigned a randomly generated item number. Manufacturing batch records for production of each of the five supplements were reviewed by a trained, independent expert in dietary supplement quality control and assurance (C.M.L.) before approval for use within the present study. All supplements were independently validated for nutritional facts and total amino acids using validated, approved methods at Covance Laboratories, Inc. (Madison, WI, USA), a pre-qualified third-party analytical laboratory, and results reviewed by C.M.L. prior to the supplements being approved for use within the present study. Once analysis was complete, a Lockwood, LLC representative not involved in the study released the code for all treatments. 

### 2.8. Nutritional Intake Monitoring

Participants were instructed to maintain their normal dietary habits along with returning a 4-day food log (2 week days and both weekend days) at baseline (T1), week 6 (T20) and week 12 (T39). On each occasion, participants were given detailed written and verbal instructions on completing the food logs. Dietary intake data were analyzed using the open-sourced software myfitnesspal (MyFitnessPal, Inc., San Francisco, CA, USA), which has been employed to analyze food intake data in other studies [42,43,44,45,46,47,48]. 

### 2.9. Immunofluorescent Histochemistry for Muscle Fiber Type-Specific Characteristics

Muscle sections were analyzed for type I fiber cross sectional area (CSA), type II fiber CSA, type I fiber myonuclear number, type II fiber myonuclear number, and total (non-fiber type-specific) satellite cell number. Methods for immunofluorescent histochemistry have been employed previously in our laboratory and described elsewhere [25,49]. Briefly, sections from OCT-preserved samples were cut at a thickness of 20 μm using a cryotome (Leica Biosystems; Buffalo Grove, IL, USA) and were adhered to positively-charged histology slides. Once all samples were sectioned, batch processing occurred for immunofluorescent histochemistry. During batch processing, sections were air-dried at room temperature for 30 min, fixed with 10% formalin for 10 min, permeabilized in a phosphate-buffered saline (PBS) solution containing 0.5% Triton X-100, and blocked with 100% Pierce Super Blocker (Thermo Fisher Scientific; Waltham, MA, USA) for 25 min. 

For fiber type staining (following blocking), sections were subsequently washed for 5 min in PBS and incubated for 1 h with a primary antibody solution containing rabbit anti-dystrophin IgG (Thermo Fisher Scientific; 10 μL antibody per 1 mL of blocking solution) and mouse anti-myosin II IgG (catalog #: SC71; Hybridoma Bank; 100 μL per 1 mL of blocking solution). Sections were then washed for 5 min in PBS and incubated in the dark for 1 h with a secondary antibody solution containing Texas Red-conjugated anti-rabbit IgG (Vector Laboratories; Burlingame, CA, USA), and Alexa Fluor 488-conjugated anti-mouse IgG (Thermo Fisher Scientific) (10 μL of all secondary antibodies per 1 mL of blocking solution). Sections were then washed for 5 min in PBS, air-dried and were mounted with fluorescent media containing 4,6-diamidino-2-phenylindole (DAPI; Vector Laboratories). Following mounting, slides were stored in the dark at 4 °C until immunofluorescent images were obtained.

For satellite cell staining (following blocking), separate sections were incubated for 1 h with a pre-diluted commercially-available primary antibody solution containing rabbit anti-dystrophin IgG (Thermo Fisher Scientific), and 1:15 dilution of mouse anti-Pax7 IgG (Hybridoma Bank, Iowa City, IA, USA) for 1 h. Sections were then washed for 5 min in 1× PBS and incubated in the dark for 1 h with a secondary antibody solution containing 1:100 dilution of Texas Red-conjugated anti-rabbit IgG (Vector Laboratories) and Alexa Fluor 488-conjugated anti-mouse IgG (Thermo Fisher Scientific). Sections were then washed for 5 min in PBS, air-dried and were mounted with fluorescent media containing DAPI (Vector Laboratories). Following mounting, slides were stored in the dark at 4 °C until immunofluorescent images were obtained. 

After staining was performed on all sections, digital images were captured using a fluorescence microscope (Nikon Instruments; Melville, NY, USA) and 20× objective. Approximate exposure times were 600 ms for red and green imaging and 30 ms for blue imaging. For fiber typing, our staining method allowed the identification of cell membranes (detected by the Texas Red filter), type II fiber green cell bodies (detected by the FITC filter), type I fiber black cell bodies (unlabeled), and myonuclei (detected by the DAPI filter). For satellite cell identification, our staining method allowed the identification of cell membranes (detected by the Texas Red filter), small green cell bodies as satellite cells (detected by the FITC filter), and myonuclei (detected by the DAPI filter). Measurements of type II fiber cross sectional area (CSA) were performed using the open-sourced software CellProfiler^TM^ [50] per modified methods previously described whereby the number of pixels counted within the border of each muscle fiber were converted to a total area in microns-squared (μm^2^). Measurements of fiber type-specific myonuclear number were also performed using open-sourced software CellProfiler^TM^ to discriminate the fiber border that corresponded to each myonuclei. Satellite cells were manually counted using a grid function in the NIS Elements software (Nikon Instruments) and handheld tally counter. Per the recommendations of Mackey et al. [51], at least 50 fibers per specimen were quantified to obtain accurate CSA, myonuclear number and satellite cell values.

### 2.10. SQ Fat CSA Analysis

As mentioned above, gluteal fat was obtained at T1 and T39 for SQ fat analysis. Following tissue processing and H&E staining, SQ fat CSA analysis was performed as previously published by our laboratory [52,53]. Briefly, SQ fat samples were removed from formalin and then washed in cold running tap water, embedded, and stored in 70% alcohol. Dehydration was accomplished by gradually increasing percentages of ethyl alcohol to replace the water content in the tissue. Hemo-De was subsequently used to clear the tissue from the ethyl alcohol to allow infiltration with paraffin. The paraffin tissue blocks were sectioned into 6 μm slices and placed onto glass microscope slides. Paraffin was removed with xylene, the mounted sections were stained with hematoxylin and eosin, and sample sections were enclosed with a coverslip and mounting media. Two 10× objective digital images per sample were obtained using bright-field imaging (Nikon Instruments), and CSAs were obtained from at least 50 adipocytes per image using ImageJ (National Institutes of Health; Bethesda, MD, USA).

### 2.11. A Priori Sample Size Calculations and Statistical Analyses

Based upon meta-data compiled by Phillips [14], whey protein-supplemented subjects participating in resistance training for at least 8 weeks experienced, on average, an estimated 3.0 ± 0.6 kg increase in muscle mass, while those supplementing with soy protein experienced a ~1.4 ± 0.3 kg increase and those supplementing with a carbohydrate-based placebo presented a ~1.0 ± 0.2 kg increase. To obtain an adequately-powered sample-size for each treatment, a priori calculations (non-centrality parameter = 2.8, power = 0.80, pooled standard deviation values of 0.5) suggested that a sample-size of 3 participants per group would be needed to detect a significant difference between whey protein versus soy and/or other potential treatments. However, in order to sufficiently power the trial, we attempted to enroll 15–20 subjects per treatment.

All data are presented in tables and figures as means ± standard error of the mean (SE) values. Statistics were performed using SPSS v22.0 (IBM; Armonk, NY, USA) and Microsoft Excel when applicable. A Shapiro–Wilk’s test was employed for all dependent variables to test for distribution normality. If values were not normally distributed then values were square root-transformed and re-tested using Shapiro–Wilk’s tests to ensure that values were normally distributed. All raw and transformed dependent variables (except nutrition data) were then compared between treatment groups using 5*2 group*time (G*T) two-way repeated measures analysis of covariance (ANCOVA) tests with T1 values for each respective dependent variable serving as the covariate. If a significant time effect was present, then within-group dependent samples t-test were performed between T1 and T39 values. If a significant G*T interaction was present, within-group dependent samples *t*-tests were performed between T1 and T39 values, and one-way analysis of variance (ANOVA) tests with Tukey post hoc tests were performed at the T39 time point. All nutritional dependent variables were compared between treatment groups using 5*3 (group*time) two-way repeated measures ANCOVAs with T1 values for each respective dependent variable serving as the covariate. If a significant time effect was present, then within-group pairwise comparisons were performed using Bonferroni post hoc tests. If a significant group*time interaction was present, then within-group dependent-samples *t*-tests were performed between T1 and T20 as well as T39 values, and one-way ANOVAs with Tukey post hoc tests were performed at the T20 and T39 time points.

## 3. Results

### 3.1. Subject Compliance and Baseline Characteristics

**Figure 2:**
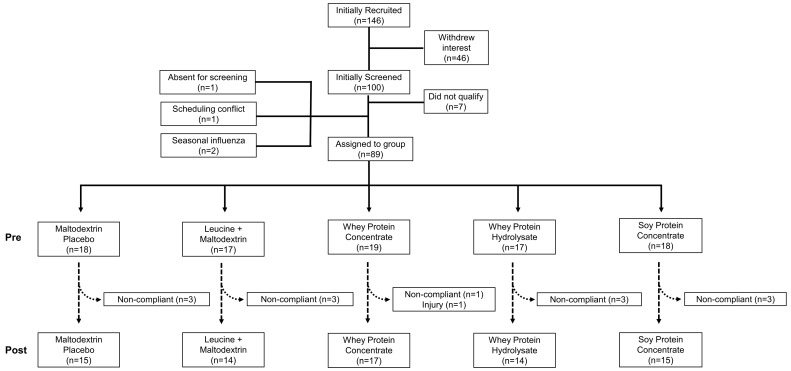
CONSORT diagram of the study. Legend: Details regarding this Consolidated Standards of Reporting Trails (CONSORT) diagram can be found in the results.

The Consolidated Standards of Reporting Trials (CONSORT) diagram for this study is presented in Figure 2. Briefly, a total of 146 potential participants were recruited for the study. Of these individuals, 46 withdrew interest and 100 were pre-screened. Of these 100 individuals, four did not consent due to scheduling conflicts or illness, and seven did not qualify for the study. Of the 89 participants that provided consent and began the study, a total of 75 successfully completed the intervention (PLA *n* = 15, LEU *n* = 14, WPC *n* = 17, WPH *n* = 14, and SPC *n* = 15). Notably, 13 participants were removed from the study due to lack of compliance with supplementation or resistance training. One subject in the WPC group had to withdraw from the study due to a musculoskeletal injury sustained during training which was reported to the Auburn University IRB.

There were no baseline differences between supplement groups for select dependent variables related to age, body composition, or strength (see Table 3 for *p*-values). Overall, supplement compliance was 95% and did not differ between groups (ANOVA, *p* = 0.203) Furthermore, overall training compliance was 94% and did not differ between groups (ANOVA, *p* = 0.296). 

### 3.2. Self-Reported Nutritional Intakes

All self-reported food intakes during the intervention are reported in Table 4. Caloric and macronutrient intakes (i.e., total and relative calories, protein, carbohydrates and fats) did not differ between groups at T1 (all ANOVA *p*-values > 0.50). Furthermore, a significant main effect of time for Caloric intake existed whereby T20 and T39 were greater than T1 (*p* < 0.001; Table 4). However, there was no G*T interaction (*p* = 0.847). 

A significant main effect of time existed for total daily protein intake whereby T20 and T39 was greater than T1 (*p* < 0.001; Table 4). Additionally, there was a G*T interaction (*p* < 0.001) whereby (a) WPC/WPH/SPC ingested more protein at T20 and T39 relative to T1 (*p* < 0.001); (b) LEU ingested more protein at T39 relative to T1 (*p* < 0.01); (c) at T20 WPC/WPH/SPC > LEU/PLA (*p* < 0.01) and SPC > WPC (*p* < 0.01); and (d) at T39 WPC/WPH/SPC > LEU/PLA (*p* < 0.05). A significant main effect of time also existed for relative protein (body mass-adjusted) intake whereby T20 and T39 was greater than T1 (*p* < 0.001; Table 4). Additionally, there was a G*T interaction (*p* < 0.001) whereby (a) WPC/WPH/SPC ingested more protein at T20 and T39 relative to T1 (*p* < 0.001); (b) LEU ingested more protein at T39 relative to T1 (*p* < 0.05); (c) at T20 WPC/WPH/SPC > LEU/PLA (*p* < 0.05); and (d) at T39 WPC/SPC > LEU/PLA (*p* < 0.05). 

A significant main effect of time existed for total daily carbohydrate intake whereby T20 and T39 was greater than T1 (*p* < 0.001; Table 4). There was also a G*T interaction (*p* < 0.001) whereby (a) PLA/LEU/WPH/SPC ingested more carbohydrates at T20 and T39 relative to T1 (*p* < 0.05); (b) WPC ingested more carbohydrates at T39 relative to T1 (*p* < 0.05); (c) at T20 PLA > WPC/WPH/SPC (*p* < 0.05); and (d) at T39 PLA > WPC/WPH/SPC (*p* < 0.05). A significant main effect of time also existed for relative (body mass-adjusted) carbohydrate intake whereby T20 and T39 was greater than T1 (*p* < 0.001; Table 4). Additionally, there was a G*T interaction (*p* < 0.01) whereby (a) PLA/LEU ingested more carbohydrates at T20 and relative to T1 (*p* < 0.01); (b) WPH/SPC ingested more carbohydrates at T39 relative to T1 (*p* < 0.05); (c) at T20 LEU > WPC/SPC (*p* < 0.05); and (d) at T39 PLA > WPC (*p* < 0.05).

A significant main effect of time existed for total daily fat intake whereby T20 and T39 was greater than T1 (*p* < 0.001). However, there was no G*T interaction for total daily fat intake or relative (body mass-adjusted) fat intake (*p* = 0.549 and *p* = 0.809, respectively). 

### 3.3. Training Volume, 3-RM Strength, IMTP

**Figure 3:**
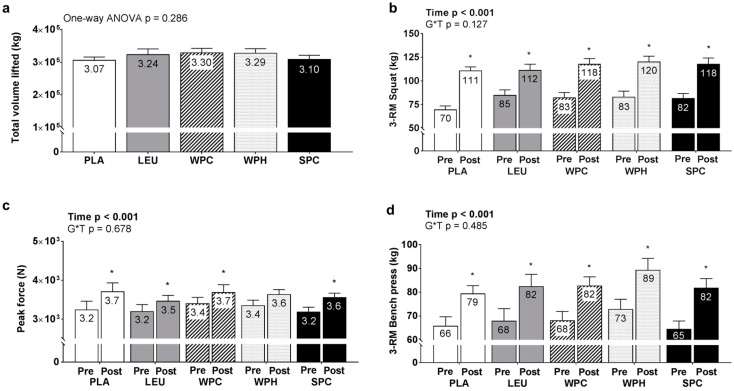
Total volume lifted and changes in strength measures between groups. Legend: Data include total volume lifted during the 12-week training intervention (**panel a**) as well as pre- and post-intervention 3-repetition maximum (RM) squat values (**panel b**), 3-RM bench press values (**panel c**), and isometric mid-thigh pull (IMTP) peak force values (**panel d**). Each bar graph depicts group averaged data presented as mean + standard error values, and mean values are presented within each bar. Additional abbreviations: PLA, maltodextrin placebo; LEU, L-leucine; WPC, whey protein concentrate; WPH, whey protein hydrolysate; SPC, soy protein concentrate; Symbol: * within-group increase from pre- to post training (*p* < 0.05).

Training volume during the intervention did not differ between groups (ANOVA, *p* = 0.286; Figure 3a). Significant main effects of time existed for 3-RM squat (*p* < 0.001; Figure 3b), 3-RM bench press (*p* < 0.001; Figure 3c) and IMTP (*p* < 0.001; Figure 3d) whereby T39 values were greater than T1 values. However, no significant G*T interactions existed for these variables (*p* = 0.127, 0.485, and 0.684 for 3-RM squat, 3-RM bench press, and IMTP, respectively).

### 3.4. Changes in Body Mass, TBMM, Fat Mass, and Vastus Lateralis Muscle Thickness between Groups

**Figure 4:**
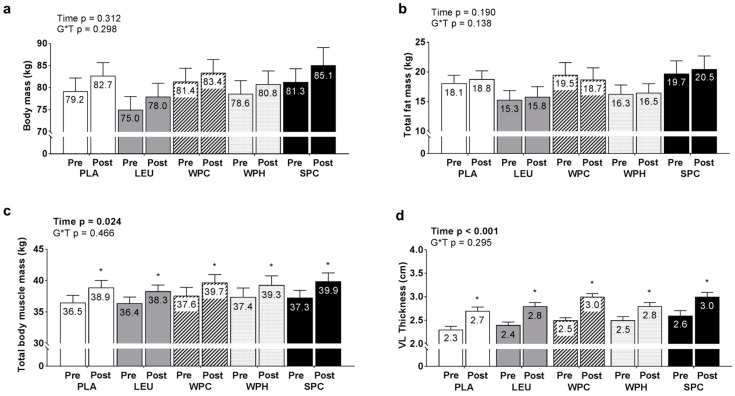
Changes in body composition variables and vastus lateralis muscle thickness between groups. Legend: Data include pre- and post-intervention body mass values (**panel a**), total fat mass values determined by dual x-ray absorptiometry (DXA; **panel b**), total body muscle mass (TBMM) values determined by DXA (**panel c**), and vastus lateralis (VL) thickness determined by ultrasonography (**panel d**). Each bar graph depicts group averaged data presented as mean + standard error values, and mean values are presented within each bar. Additional abbreviations: PLA, maltodextrin placebo; LEU, L-leucine; WPC, whey protein concentrate; WPH, whey protein hydrolysate; SPC, soy protein concentrate; Symbol: * within-group increase from pre- to post training (*p* < 0.05).

No significant main effects of time or G*T interactions existed for changes in total body mass (Figure 4a) or fat mass (Figure 4b). Significant main effects of time did exist for changes in TBMM (*p* < 0.001; Figure 4c) and vastus lateralis muscle thickness (*p* < 0.001; Figure 4d) whereby T39 values were greater than T1 values. However, there were no G*T interactions for these variables (*p* = 0.847 and 0.295 for TBMM and vastus lateralis muscle thickness, respectively). 

### 3.5. Changes in Fiber Type-Specific CSA and Myonuclear Number as Well as Total Satellite Cell Number between Groups

**Figure 5:**
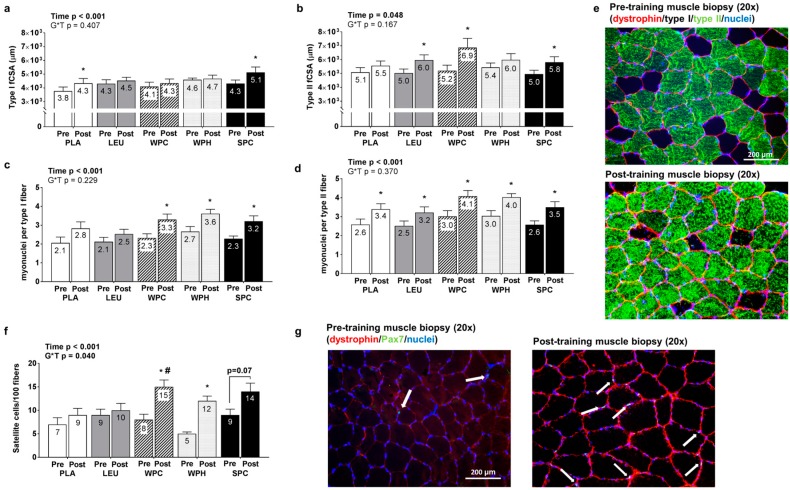
Changes in muscle fiber cross sectional area, myonuclear number and satellite cell counts between groups. Legend: Data include pre- and post-intervention type I and type II fiber cross sectional area (CSA) values (**panels a**,**b**), type I and type II fiber myonuclear number values (**panels c**,**d**), and total satellite cell counts (**panel f**). Due to poor tissue quality on select subjects, n-sizes were as follows: PLA *n* = 13, LEU *n* = 13, WPC *n* = 15, WPH *n* = 12, and SPC *n* = 14. Representative 20× objective histology images from one subject demonstrating myofiber hypertrophy and increases in satellite cell counts are presented in (**panels e**,**g**), respectively. Each bar graph depicts group averaged data presented as mean + standard error values, and mean values are presented within each bar. Abbreviations: PLA, maltodextrin placebo; LEU, L-leucine; WPC, whey protein concentrate; WPH, whey protein hydrolysate; SPC, soy protein concentrate. Symbols: * within-group increase from pre- to post training (*p* < 0.05); #, WPC > PLA at T39 (*p* < 0.05).

Significant main effects of time existed for changes in type I fiber CSA (*p* < 0.001; Figure 5a) and type II fiber CSA (*p* = 0.048; Figure 5b) whereby T39 values were greater than T1 values, although no G*T interactions for these variables existed (*p* = 0.407 and *p* = 0.167, respectively). Significant main effects of time also existed for changes in type I fiber myonuclear number (*p* < 0.001; Figure 5c) and type II fiber myonuclear number (*p* < 0.001; Figure 5d) whereby T39 values were greater than T1 values, although no G*T interactions for these variables existed (*p* = 0.370 and 0.229 for type I and II fiber myonuclear number, respectively). A significant main effect of time existed for changes in total satellite cell counts whereby T39 was greater than T1 (*p* < 0.001; Figure 5f). Additionally, there was a G*T interaction (*p* < 0.05) whereby (a) WPC and WPH prompted more satellite cells at T39 relative to T1 (*p* < 0.05) and (b) WPC expressed a significantly greater number of satellite cells than PLA at T39 (*p* = 0.033).

### 3.6. Changes in SQ Adipocyte CSA between Groups

**Figure 6:**
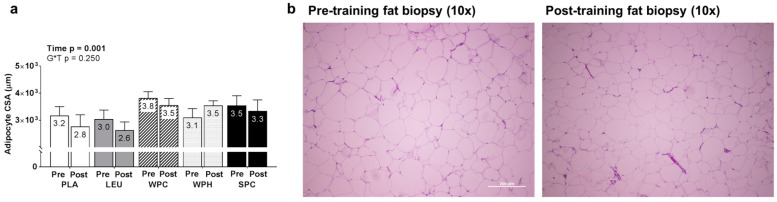
Changes in gluteal subcutaneous adipocyte cross sectional area between groups. Legend: Pre- and post-training subcutaneous adipocyte cross sectional area (CSA) values are presented in (**panel a**). Representative 10x objective histology images from one subject demonstrating a reduction in fat cell size is presented in (**panel b**). Due to poor tissue quality on select subjects, n-sizes were as follows: PLA *n* = 14, LEU *n* = 12, WPC *n* = 14, WPH *n* = 10, and SPC *n* = 13. The bar graph depicts group averaged data presented as mean + standard error values, and mean values are presented within each bar. Abbreviations: PLA, maltodextrin placebo; LEU, L-leucine; WPC, whey protein concentrate; WPH, whey protein hydrolysate; SPC, soy protein concentrate.

A significant main effect of time for adipocyte CSA existed whereby T39 was less than T1 (*p* = 0.001; Figure 6). However, within-group dependent sample *t*-tests did not reveal any significant effect for time between T1 and T39 within groups (all *p*-values > 0.200). Likewise, no G*T interaction existed (*p* = 0.250). 

## 4. Discussion

We sought to determine the effects of LEU or different protein supplements standardized to ~3.0 g LEU, consumed twice daily, on changes in body composition, strength, and histological changes in skeletal muscle and adipose tissue attributes in previously untrained, college-aged males when combined with 12 weeks of resistance training. The main findings for our study included the following: (a) there was a training effect, but no effect of supplementation, for increases in TBMM, strength (i.e., IMTP, 3-RM squat, 3-RM bench press), vastus lateralis muscle thickness, and type I and II fiber CSA, type I and II fiber myonuclear number (b) WPC and WPH, but not LEU or PLA, significantly increased satellite cell counts, and increases in the SPC group approached significance, and (c) there was a time/training effect for decrements in SQ fat cell size (*p* = 0.001).

Contrary to our hypotheses, our data indicated that there was a training effect, but no supplementation effect, on increases in TBMM, vastus lateralis thickness, and type I/II fiber CSA values. Several studies have demonstrated that whey protein supplementation during resistance training increases indices of muscle anabolism compared to placebo [1,2,3,4,54,55,56] or soy [5] supplementation. However, our findings are in agreement with other literature reporting that protein supplementation (i.e., WPC/WPH/SPC) provides no added benefit to increasing muscle mass when consumed over an 8 to 16 week resistance training period [6,9,13,57,58,59,60]. Our null findings may have been due to a variety of factors. For instance, younger males have been reported to experience robust hypertrophic responses to resistance exercise when compared to middle-aged and older males and females (younger and older) over the first 4 months of training [61]. Hence, many of our null findings could be attributed to our examining the effects of these supplements in younger, untrained males who seemingly respond the most favorably to resistance training. We also posit that our training model was very advanced for novice lifters (i.e., 30 sets/week for upper and lower body muscles). Consequently, the employed training model could have obscured any additive anabolic effects that additional LEU or protein supplementation may have otherwise provided. It is also notable that all groups reported increasing Calorie intakes ~600–800 kcal/day from T1 to T39 (time *p* < 0.001), and all groups consumed at least 1.1 g/kg/day of protein at T1 and 1.3 g/kg/day at T39. Thus, in lieu of hypotheses put forth by Hoffman et al. [62] suggesting that 1.2 g/kg/day of protein is adequate to support muscle anabolism with resistance training, we posit that all of the participants herein were in adequately-fed states throughout the study and may minimally benefit from additional LEU or protein supplementation. Finally, given that others have reported that whey protein enhances muscle anabolism in resistance trained individuals [1,2], along with evidence indicating that trained individuals require additional protein intake to maintain a net neutral protein balance [63,64,65], it is plausible that whey protein supplementation may only benefit those that undergo more prolonged, strenuous resistance training.

Satellite cells have the capacity to divide and fuse to pre-existing muscle fibers in order to promote further increases in muscle fiber growth [66,67,68,69,70]. Furthermore, it has been suggested that resistance training-induced increases in satellite cell number are obligatory for skeletal muscle hypertrophy to occur in humans [71]. Interestingly, relative to T1, the WPC and WPH protein supplemented groups experienced significant increases in satellite cell counts at T39, whereas the SPC group trended towards significance (*p* = 0.07) and the LEU and PLA groups did not exhibit this effect. Collectively, our data suggesting that whey protein, rather than LEU, stimulates myogenesis are in agreement with Olsen et al. [72] who reported a 50% increase in total satellite cell number following 16 weeks of strength training and whey protein supplementation. Likewise, Farup et al. [73] reported a 132% and 78% increase in type II and type I fiber satellite cell number, respectively with 12 weeks of concentric exercise and whey protein supplementation. While the mechanisms of action were not directly examined herein or in any of the abovementioned studies, it is notable that Hulmi et al. [4] reported a 120% increase in cdk2 mRNA expression (a regulator of satellite cell proliferation) following 21 weeks of resistance training and whey protein supplementation. Similarly, Roberts et al. [68] reported that whey protein supplementation prior to one bout of lower body resistance exercise in younger males robustly increased MyoD mRNA expression levels 6 h following exercise which potentially indicated an increase in satellite cell activation. Thus, it is possible that protein supplementation, or increasing protein intake levels in general, may up-regulate genes within satellite cells responsible for enhanced proliferation in an acute and chronic manner which act to increase satellite cell number. Other evidence has also suggested that matrix metalloprotease (MMP) enzymes stimulate satellite cell activation and migration [74]. In this regard, our group has previously reported that the WPH utilized herein possesses MMP2/9 activity [75]. Moreover, others have reported that a variety of proteins and enzymes are contained within dairy-derived exosomal cargos [76], and dairy-derived exosomes can traverse the digestive system and target numerous tissues in vivo [77]. Hence, it also remains possible that whey protein-derived MMPs can traverse the digestive system via exosomal cargos to stimulate satellite cell activity. It should be noted, however, that a more recent investigation by Reidy et al. [7] indicated that whey or dairy-soy protein blend supplementation did not enhance satellite cell number following 12 weeks of resistance training. Thus, more data are needed to examine how increasing dietary protein intake mechanistically affects satellite cell turnover and if protein-induced increases in satellite cell number provide any added benefit to resistance-trained individuals (i.e., reducing recovery time between training bouts due to satellite cell-mediated recovery mechanisms).

This study is unique in that it is the first study to examine how LEU or protein supplementation affects SQ adipocyte CSA values. Although a secondary aim, our rationale for performing these analyses was due to our prior work which has demonstrated that WPH supplementation acutely increases lipolysis markers in rodents [18,71] and reduces fat mass in younger males following 10 weeks of resistance training [78]. While mechanisms for these prior findings have not been characterized, we have previously posited that the lipolytic effects observed with WPH supplementation may be due to unidentified peptides (produced during the hydrolysis manufacturing process) being absorbed from the digestive system and acting as ligands for fat cell membrane receptors [71]. Notwithstanding, we report that, while there was a training effect for the reduction in SQ adipocyte CSA levels (−210 μm^2^; time *p* = 0.001), WPH supplementation did not affect total body fat mass or SQ fat CSA values. It is difficult to reconcile the discrepancies between studies, and our hypotheses regarding WPH supplementation and SQ fat histological attributes require more research.

## 5. Experimental Considerations

One notable limitation to the current study is the relatively small sample size per group (*n* = 14–17), and this limitation was primarily due to resource constraints rather than faulty experimental design. In this regard, others have suggested that ≥20 subjects per group are needed in order to determine a significant between-treatment effect regarding protein supplementation and changes in muscle mass [5,79,80]. A second limitation was the relatively shorter intervention time (i.e., 12 weeks) employed for the current study. Limited evidence exists regarding the anabolic effects of resistance training with protein supplementation over a ≥6 month period [5]. Thus, implementing the current study design with more sampling time points and larger group sizes is warranted. Finally, we posit that the age and gender of our studied population limits the scope of our conclusions as per our discussion above regarding the robust responses that younger males in well-fed states typically exhibit in response to resistance training. Thus, more research is needed on replicating the current study design in older males and younger and older female participants.

## 6. Conclusions

In conclusion, our study demonstrates that neither LEU nor protein supplementation (standardized to LEU) in previously untrained, college-aged males provide added benefit in increasing whole-body skeletal muscle mass or whole-body strength. We do report, however, that whey protein supplementation significantly increases skeletal muscle satellite cell number with resistance training; this being a finding that requires further elucidation. 

## Figures and Tables

**Table 1:** Training load progression.

Week	Training Paradigm	
0	Days 1–3	Familiarization session, IMTP and 3-RM Testing
1	Day 1: 4 × 10	51% of Est. 1-RM
	Day 2: 6 × 4	60% of Est. 1-RM
	Day 3: 5 × 6	56% of Est. 1-RM
2	Day 1: 4 × 10	60% of Est. 1-RM
	Day 2: 6 × 4	70% of Est. 1-RM
	Day 3: 5 × 6	65% of Est. 1-RM
3	Day 1: 4 × 10	70% of Est. 1-RM
	Day 2: 6 × 4	79% of Est. 1-RM
	Day 3: 5 × 6	74% of Est. 1-RM
4	Day 1: 4 × 10	73% of Est. 1-RM
	Day 2: 6 × 4	89% of Est. 1-RM
	Day 3: 5 × 6	84% of Est. 1-RM
5	Day 1: 4 × 10	78% of Est. 1-RM
	Day 2: 6 × 4	95% of Est. 1-RM
	Day 3: 5 × 6	90% of Est. 1-RM
6	Day 1: 4 × 10	82% of Est. 1-RM
	Day 2: 6 × 4	100% of Est. 1-RM
	Day 3: 5 × 6	94% of Est. 1-RM
7	Day 1–3: 4 × 5 (de-load)	60% of Est. 1-RM
8	Day 1: 4 × 10	74% of Est. 1-RM
	Day 2: 6 × 4	90% of Est. 1-RM
	Day 3: 5 × 6	85% of Est. 1-RM
9	Day 1: 4 × 10	83% of Est. 1-RM
	Day 2: 6 × 4	101% of Est. 1-RM
	Day 3: 5 × 6	96% of Est. 1-RM
10	Day 1: 4 × 10	87% of Est. 1-RM
	Day 2: 6 × 4	107% of Est. 1-RM
	Day 3: 5 × 6	98% of Est. 1-RM
11	Day 1: 4 × 10	90% of Est. 1-RM
	Day 2: 6 × 4	109% of Est. 1-RM
	Day 3: 5 × 6	102% of Est. 1-RM
12	Day 1–2: 4 × 5 (de-load)	60% of Est. 1-RM
	Day 3: IMTP and 3-RM Testing	108% of Est. 1-RM

Legend: Estimated one repetition maximum (Est. 1-RM) was calculated per the NSCA’s recommended guidelines (i.e., 3-RM/0.93). Abbreviations: IMTP, isometric mid-thigh pull; 3-RM three repetition maximum; 1-RM, one repetition maximum; NSCA, National Strength and Conditioning Association.

**Table 2:** Nutritional components per serving for the different supplements.

Variable	PLA	LEU	WPC	WPH	SPC
Calories	204	200	184	192	266
Total Fat (g)	2.8	2.0	3.5	4.6	4.5
Saturated Fat (g)	2.3	1.6	2.3	3.3	2.6
Trans Fat (g)	0.0	0.0	0.1	0.2	0.2
Cholesterol (mg)	3.8	2.9	74.0	74.3	5.3
Total Carbohydrate (g)	44.4	43.1	12.0	12.2	17.2
Dietary Fiber (g)	1.6	1.8	1.8	2.2	1.5
Sugars (g)	6.0	5.1	5.9	3.2	6.2
Protein (g)	0.4	2.3	26.3	25.4	39.2
Alanine (mg)	7	7	1397	1430	1646
Arginine (mg)	8	8	766	773	2969
Aspartic Acid (mg)	15	16	2881	3010	4537
Cystine (mg)	0	0	651	728	536
Glutamic Acid (mg)	36	35	4530	4730	7154
Glycine (mg)	6	6	489	543	1597
Histidine (mg)	0	0	470	477	910
Isoleucine (mg)	8	28	1736	1820	1842
Leucine (mg)	15	2871	2794	2910	2960
Lysine (mg)	11	79	2386	2640	2362
Methionine (mg)	0	8	598	611	540
Phenylalanine (mg)	8	9	861	908	1980
Proline (mg)	14	13	1630	1670	2029
Serine (mg)	10	9	1348	1400	1950
Threonine (mg)	7	7	1853	1900	1499
Tryptophan	0	0	482	525	501
Tyrosine (mg)	7	7	808	839	1480
Valine (mg)	11	14	1465	1530	1754
Total EAAs (mg)	60	3016	12,645	13,321	14,348
Total BCAAs (mg)	34	2913	5995	6260	6556
Calcium (mg)	15	15	155	152	165
Iron (mg)	0.38	0.35	0.63	1.04	5.21
Potassium (mg)	32	37	230	464	961
Sodium (mg)	91	105	133	310	217
Vitamin D3 (IU)	0.0	0.0	0.0	0.0	0.0
Degree of hydrolysis (%)	N/A	N/A	N/A	12.5	N/A
M.W. range (%)					
>10.0 kD	-	-	74.3	29.0	86.0
5.0–10.0 kD	-	-	5.1	5.3	3.6
2.0–5.0 kD	-	-	15.4	10.2	2.6
1.0–2.0 kD	-	-	1.6	10.8	1.2
0.5–1.0 kD	-	-	0.9	15.5	0.9
<0.5 kD	-	-	2.7	29.3	5.6

Abbreviations: PLA, maltodextrin placebo; LEU, L-leucine; WPC, whey protein concentrate; WPH, whey protein hydrolysate; SPC, soy protein concentrate; g, grams; mg, milligrams; IU, international units; kD, kilodaltons; N/A, not applicable.

**Table 3:** Baseline characteristics between groups.

Variable	PLA (*n* = 15)	LEU (*n* = 14)	WPC (*n* = 17)	WPH (*n* = 14)	SPC (*n* = 15)	ANOVA *p*-Value
Age (years)	21 ± 1	20 ± 1	21 ± 1	21 ± 1	21 ± 1	0.811
Height (cm)	183 ± 2	179 ± 1	179 ± 2	182 ± 2	182 ± 2	0.454
Body Mass (kg)	79 ± 3	75 ± 2	81 ± 3	79 ± 3	81 ± 3	0.600
Lean body mass (kg)	58 ± 4	57 ± 3	59 ± 4	59 ± 5	59 ± 4	0.899
Total Fat Mass (kg)	18 ± 3	15 ± 3	19 ± 5	16 ± 3	20 ± 5	0.378
Strength 3-RM (kg)						
Squat	70 ± 8	83 ± 12	82 ± 11	79 ± 14	82 ± 10	0.369
Bench press	66 ± 8	67 ± 11	68 ± 8	73 ± 9	65 ± 7	0.650
IMTP (N)	3247 ± 215	3205 ± 170	3476 ± 141	3461 ± 130	3192 ± 117	0.488

Legend: Values are presented as means ± SE. Abbreviations: PLA, maltodextrin placebo; LEU, L-leucine; WPC, whey protein concentrate; WPH, whey protein hydrolysate; SPC, soy protein concentrate; 3-RM, 3-repetition maximum; IMTP, isometric mid-thigh pull; cm, centimeters kg, kilograms; N, newtons.

**Table 4:** Self-reported nutrient intakes between groups.

Variable	Baseline	Week 6	Week 12	ANCOVA *p*-Values
Group				
Energy intake (kcal/day)				Time *p* < 0.001 G*T *p* = 0.865
PLA	2109 ± 166	2756 ± 236 *	2812 ± 232 *	
LEU	1835 ± 116	2303 ± 165 *	2488 ± 132 *	
WPC	1866 ± 115	2305 ± 116 *	2389 ± 177 *	
WPH	2039 ± 149	2611 ± 156 *	2617 ± 144 *	
SPC	1853 ± 136	2461 ± 129 *	2611 ± 158 *	
Protein intake (g/day; g/kg/day)				
PLA	94 ± 7; 1.2 ± 0.1	109 ± 8^c^; 1.3 ± 0.1 ^b^	111 ± 11^b^; 1.3 ± 0.1 ^b^	Time *p* < 0.001 G*T *p* < 0.001
LEU	87 ± 6; 1.2 ± 0.1	96 ± 8^c^; 1.3 ± 0.1 ^b^	108 ± 10 *^,b^; 1.4 ± 0.1 ^b^	
WPC	88 ± 6; 1.1 ± 0.1	142 ± 5 *^,a^; 1.8 ± 0.1 *^,a^	145 ± 6 *^,a^; 1.8 ± 0.1 *^,a,b^	
WPH	94 ± 8; 1.2 ± 0.1	160 ± 7 *^,a,b^; 2.0 ± 0.1 *^,a^	153 ± 7 *^,a^; 1.9 ± 0.1 *^,a^	
SPC	88 ± 6; 1.1 ± 0.1	176 ± 7 *^,b^; 2.1 ± 0.1 *^,a^	179 ± 10; 2.1 ± 0.1 *^,a^	
Carbohydrate intake (g/day; g/kg/day)				
PLA	244 ± 23; 3.1 ± 0.3	337 ± 21 *^,a^; 4.2 ± 0.3 *^,a,b^	348 ± 29 *^,a^; 4.2 ± 0.3 *^,a^	Time *p* < 0.001 G*T *p* = 0.002
LEU	206 ± 17; 2.8 ± 0.3	303 ± 24 *^,a,b^; 4.0 ± 0.4 *^,a^	310 ± 21 *^,a,b^; 4.1 ± 0.4 *^,a,b^	
WPC	215 ± 14; 2.8 ± 0.3	231 ± 18^b^; 2.9 ± 0.3 ^b^	244 ± 16 *^,b^; 3.0 ± 0.2 ^b^	
WPH	208 ± 20; 2.7 ± 0.3	247 ± 15 *^,b^; 3.1 ± 0.3 *^,a,b^	255 ± 16 *^,b^; 3.3 ± 0.3 *^,a,b^	
SPC	203 ± 18; 2.6 ± 0.3	238 ± 16 *^,b^; 2.9 ± 0.2 ^b^	256 ± 20 *^,b^; 3.1 ± 0.3 *^,a,b^	
Fat intake (g/day; g/kg/day)				
PLA	83 ± 9; 1.1 ± 0.1	106 ± 12 *; 1.3 ± 0.1	110 ± 12 *; 1.3 ± 0.1 *	Time *p* < 0.001 G*T *p* = 0.549
LEU	73 ± 5; 1.0 ± 0.1	79 ± 5; 1.0 ± 0.1	92 ± 6; 1.2 ± 0.1	
WPC	71 ± 5; 0.9 ± 0.1	87 ± 5 *; 1.1 ± 0.1 *	93 ± 12; 1.2 ± 0.1	
WPH	81 ± 6; 1.1 ± 0.1	102 ± 9 *; 1.3 ± 0.2	101 ± 7; 1.3 ± 0.1	
SPC	73 ± 6; 0.9 ± 0.1	90 ± 7 *; 1.1 ± 0.1 *	101 ± 10 *; 1.2 ± 0.1 *	

Legend: Values are means ± SE. Symbols: *, indicate within-group increases from baseline (*p* < 0.05); values that do not share superscript (^a,b,c^) letters represent between-group significance at a given time point (*p* < 0.05). Abbreviations: PLA, maltodextrin placebo; LEU, L-leucine; WPC, whey protein concentrate; WPH, whey protein hydrolysate; SPC, soy protein concentrate; G*T, group*time interaction.
